# Contribution of Ebullition to Methane and Carbon Dioxide Emission from Water between Plant Rows in a Tropical Rice Paddy Field

**DOI:** 10.1155/2015/623901

**Published:** 2015-12-29

**Authors:** Shujiro Komiya, Kosuke Noborio, Kentaro Katano, Tiwa Pakoktom, Meechai Siangliw, Theerayut Toojinda

**Affiliations:** ^1^Graduate School of Agriculture, Meiji University, Kawasaki, Kanagawa 214-8571, Japan; ^2^School of Agriculture, Meiji University, Kawasaki, Kanagawa 214-8571, Japan; ^3^Formerly Graduate School of Agriculture, Meiji University, Kawasaki, Kanagawa 214-8571, Japan; ^4^Department of Agronomy, Kasetsart University, Kamphaeng Saen, Nakhon Pathom 73140, Thailand; ^5^Rice Gene Discovery Unit, National Center for Genetic Engineering and Biotechnology (BIOTEC), National Science and Technology Development Agency, Kasetsart University, Kamphaeng Saen, Nakhon Pathom 73140, Thailand

## Abstract

Although bubble ebullition through water in rice paddy fields dominates direct methane (CH_4_) emissions from paddy soil to the atmosphere in tropical regions, the temporal changes and regulating factors of this ebullition are poorly understood. Bubbles in a submerged paddy soil also contain high concentrations of carbon dioxide (CO_2_), implying that CO_2_ ebullition may occur in addition to CH_4_ ebullition. We investigated the dynamics of CH_4_ and CO_2_ ebullition in tropical rice paddy fields using an automated closed chamber installed between rice plants. Abrupt increases in CH_4_ concentrations occurred by bubble ebullition. The CO_2_ concentration in the chamber air suddenly increased at the same time, which indicated that CO_2_ ebullition was also occurring. The CH_4_ and CO_2_ emissions by bubble ebullition were correlated with falling atmospheric pressure and increasing soil surface temperature. The relative contribution of CH_4_ and CO_2_ ebullitions to the daily total emissions was 95–97% and 13–35%, respectively.

## 1. Introduction

Understanding the dynamics of methane (CH_4_) and carbon dioxide (CO_2_) fluxes in rice paddy fields is crucial for improving the accuracy of estimating CH_4_ and CO_2_ emissions from global rice paddy fields. In particular, flooded rice paddies are considered to be a major source of anthropogenic CH_4_. Methane emissions from rice paddies in tropical Asian countries account for 90% of global annual CH_4_ emissions from rice paddies [1, 2].

Methane produced in an anaerobic-flooded paddy soil is mainly transported to the atmosphere through the aerenchyma of rice plants [3–5]. Such emissions through the aerenchyma are estimated to account for 48–85% of net CH_4_ emissions throughout the rice-cropping season [5]. In addition, CO_2_ exchange in paddy fields mainly results from photosynthesis and respiration of rice plants, as well as soil microbial respiration.

Also, some of the CH_4_ and CO_2_ produced in rice field soil is directly emitted to the atmosphere through paddy water. In one study, when rice straw was applied to a paddy field, CH_4_ emissions via bubble ebullition from the soil accounted for 35–62% of total CH_4_ emissions [6]. However, research on the direct CH_4_ and CO_2_ exchanges between paddy soil and the atmosphere, via paddy water, is limited and so further studies are required on these emissions, as has been noted by other researchers [7, 8].

Methane in paddy soil is transported to the atmosphere through paddy water by two pathways: (1) diffusion between soil and atmosphere and (2) bubble ebullition [9]. Methane emission by bubble ebullition is considered to be greater than that by diffusion from paddy water [6]. The bubbles usually contain a high concentration of CH_4_ ranging between 1 and 82% (v/v) [10, 11] and comprise most of the total CH_4_ pool in flooded paddy soil [12]. Bubble production and ebullition are enhanced by applied organic materials during the initial plant growth period [6, 13, 14] and by organic substances originating from rice roots during later growth stages [6, 12, 14]. Although the variation of CH_4_ bubble ebullition during the cultivation period has been studied previously, the factors controlling the diurnal changes in CH_4_ ebullition remain unclear [15].

Methane ebullition from submerged peatlands, which are similar to flooded paddy soil in that they contain many bubbles, is controlled by atmospheric pressure, soil temperature, and water table level [16–19]. Falling atmospheric pressure has been shown to be the most important contributor to CH_4_ bubbling in peatlands [18, 19]. A study in rice paddy fields in Thailand also suggested that CH_4_ ebullitions occurred when atmospheric pressure dropped, but further research is needed to clarify this [20].

In contrast, CO_2_ exchange through paddy water is the result of photosynthesis of aquatic plants and respiration of both the plants and the soil microorganisms [21]. Emission due to soil respiration is suppressed by paddy water during flood irrigation [21, 22], but the CO_2_ concentration in soil bubbles is between 2.2 and 13.0% (v/v) [11, 23], which suggests that bubble ebullition will release both CH_4_ and CO_2_ from paddy soil into the atmosphere.

Therefore, in this paper, we examined the dynamics of both CH_4_ and CO_2_ ebullition in tropical rice paddy fields in Thailand using an automatically closing chamber method.

## 2. Materials and Methods

Gas field measurements were conducted on September 20th and 21st, 2014, in a rice field of Kasetsart University, Kamphaeng Saen campus (14°00′33′′N, 99°59′03′′E) located in Nakhon Pathom Province, Thailand. The soil had a clay texture (65.7% clay, 23.30% silt, and 11.0% sand) with a dry bulk density of 1.69 g m^−3^. The soil was sampled on September 17 and had a pH of 6.0 (1 : 1 for soil : water), 4.32% organic matter, 1.81% total carbon, and 1.85% total nitrogen. Seedlings of the rice variety “Homcholasit” were transplanted on June 30 at 18 × 30 cm spacing with 4-5 seedlings per hill, after the soil had been plowed on June 17 and 26 when weeds and rice plants that had grown during the fallow period were plowed into the soil. The rice plants headed on September 22 and were harvested on October 28. The paddy field was continuously flooded from June 17 until harvest, with flooding water depth maintained at 2–20 cm. During the gas measurement period, the water depth slowly decreased from 5.5 to 4 cm because there was no precipitation or irrigation.

**Figure 1:**
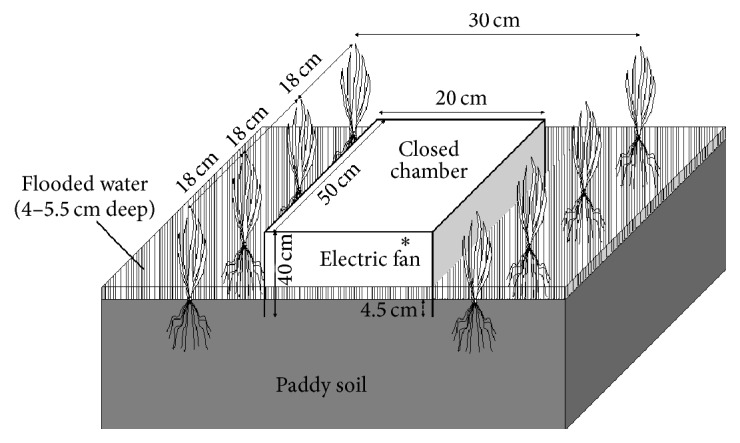
Schematic diagram of an automatic closed chamber placed between the rows of rice plants.

The CH_4_ and CO_2_ fluxes were measured using the automatic closed chamber method. A customized-bottomless polycarbonate chamber (50 × 20 cm at the base and 40 cm height, Green Blue Corp., Tokyo, Japan) was placed between the rows of rice plants on August 8; the base part was inserted 4.5 cm deep into the paddy soil (Figure 1). The lid of the chamber was automatically closed for 10 min every 1 h by a pneumatic piston, with the lid kept open for the rest of the time. A small electric fan was installed on the upper sidewall inside the chamber and was kept running throughout the experiment to uniformly mix the air within the chamber. The chamber headspace air was circulated at 500 mL min^−1^ (using a diaphragm pump; TD-4X2N, Brailsford Co., Rye, NY, USA) between the chamber and a 250 mL buffer tank placed in a shed located approximately 4 m away from the chamber to minimize the high frequency noise. A loop line was installed between the buffer tank and a wavelength-scanned cavity ring-down spectroscopy CH_4_/CO_2_ analyzer (G2201-i, Picarro Inc., Santa Clara, CA, USA). Air in the buffer tank was withdrawn to the analyzer at a flow rate of ~25 mL min^−1^ using another diaphragm pump (UN84.4 ANDC-B, KNF Neuberger Inc., NJ, USA) and then returned to the loop line. Concentrations of CH_4_ and CO_2_ were analyzed at approximately 3.6 s intervals by the gas analyzer. The sampled air was dried before entering the gas analyzer using a reflux method with a membrane dryer (SWG-A01-06, Asahi Glass Engineering Co., Chiba, Japan) so that the water vapor concentration in the air was kept <0.1%. Based on the internal volumes of the buffer tank and connecting tube and the flow rate the air inside the chamber was calculated to first reach the gas analyzer 2 min after closing the chamber lid. The measurements of CH_4_ and CO_2_ concentrations in the chamber air stopped when the chamber lid opened meaning that a measurement cycle of gas flux measurements lasted 8 min every hour.

**Figure 2:**
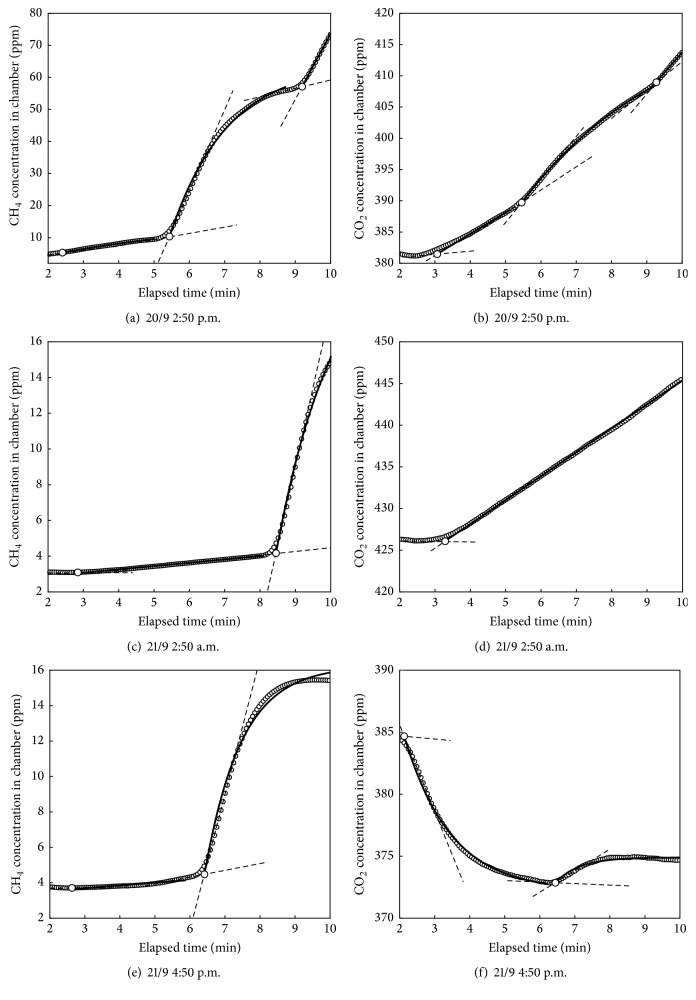
Examples of the changes in CH_4_, CO_2_ concentrations (7-point running average) in the closed chamber measured at 2:50 p.m. on September 20 ((a), (b)), at 2:50 a.m. on September 21 ((c), (d)), and at 4:50 p.m. on September 21 ((e), (f)). The solid line denotes the best fitting line for each emission/uptake. The white circle with black edge indicates the event starting point. The dashed lines denote the tangent lines at the local maximum or minimum points for CH_4_, CO_2_ emission/uptake rates, before respective increase or decrease events.

Temporal changes in CH_4_ concentration in the chamber during a measurement cycle were categorized into either a sudden increase (Figures 2(a), 2(c), and 2(e)) or a slow-constant increase (Figures 2(c) and 2(e)). Emission by bubble ebullition events was defined as a sudden increase in concentration (Δ*C*/Δ*t*) of ≥0.29 ppm min^−1^, whereas emission by diffusion was defined as a slow-constant increase (Δ*C*/Δ*t*) of <0.29 ppm min^−1^.

Changes in CO_2_ concentration in the chamber showed either an episodic increase accompanied by CH_4_ ebullition events (Figures 2(b) and 2(f)), a steady increase (Figure 2(d)), or a decrease by plant uptake (Figure 2(f)). CO_2_ emission by bubble ebullition was defined as episodic CO_2_ concentration increases accompanied by CH_4_ ebullition, whereas emission by diffusion was defined as a constant CO_2_ increase. The CO_2_ uptake by photosynthetically active aquatic plants was defined by a decrease in CO_2_ concentration (Figure 2(f)) observed during the daytime on both days.

Since CH_4_ and CO_2_ concentrations in the chamber often changed episodically with time due to bubble ebullition events (Figures 2(a), 2(b), 2(c), 2(e), and 2(f)), CH_4_ and CO_2_ fluxes were calculated for each single flux event and then summed proportionately for the time of each event to give a total flux for each 8 min measurement period. The start of each flux event was determined as the intersection between tangent lines at the inflection point of the time series of CH_4_ or CO_2_ concentrations (Figure 2). The end of each event was the time just before the start of the next flux event or the end of the 8 min measurement period (Figure 2). The gas flux *F* (mg m^−2^ h^−1^) was calculated using temporal changes in gas concentrations as [24]

(1) $$\begin{array}{c} F=\frac{V}{A}{\left[\;\frac{dC\left(t\right)}{dt}\;\right]}_{t=0}, \end{array}$$

where *V* is the headspace volume within the chamber (m^3^), *A* is the water-surface area covered by the chamber (m^2^), *t* is elapsed time (h), and *C*(*t*) is temporal changes in gas concentration (mg m^−3^) expressed as

(2) $$\begin{array}{c} C\left(\;t\;\right)=C_{max}-\left(\;C_{max}-C_{0}\;\right)\exp\left(\;-kt\;\right), \end{array}$$

where *C*_max_ is the maximum gas concentration (mg m^−3^), *C*_0_ is the initial gas concentration (mg m^−3^), and *k* is a rate constant. The values of *C*_max_, *C*_0_, and *k* were iteratively obtained using the data of observed gas concentration versus time. Substituting (2) at *t* = 0 into (1) means that the gas flux *F* (mg m^−2^ h^−1^) can be calculated as [24]

(3) $$\begin{array}{c} F=\frac{V}{A}k\left(\;C_{max}-C_{0}\;\right). \end{array}$$

Atmospheric pressure and air temperature were measured with a barometer (MPXAZ6115A and MPXV7007DP, Freescale Inc., TX, USA) and a thermometer (HMP45A, Vaisala Inc., Helsinki, Finland), respectively. Water depth in the rice field was measured with a water level sensor (eTape Continuous Fluid Level Sensor, Milone Technologies Inc., NJ, USA). Soil surface temperature was measured with a type T thermocouple.

Bubbles in soil were collected directly with a syringe by disturbing the topsoil at 3 p.m. local time on September 20. The CH_4_ and CO_2_ concentrations in the bubbles were measured using the CH_4_/CO_2_ gas analyzer after the sampled air was diluted 101 times with high-purity nitrogen gas.

## 3. Results and Discussion

### 3.1. CH_4_ Emission

Episodic and rapid increases in CH_4_ concentration were identified in 21 out of the 46 measurements (Figures 2(a), 2(c), and 2(e)). These sudden increases in CH_4_ concentration are likely to be from bubbles released from the soil to the atmosphere because the CH_4_ concentration in topsoil bubbles was as high as 63.73% v/v. In the other 25 measurements, the CH_4_ concentration in the chamber air increased gradually with time (ΔCH_4_/Δ*t* < 0.29 ppm min^−1^) during the closure period, indicating that CH_4_ was released from the water surface to the atmosphere by molecular diffusion. The CH_4_ fluxes at the water surface fluctuated between 0.7 and 218.7 mg m^−2^ h^−1^ on the observation days, which are similar to previously reported values of −0.6–192.0 mg m^−2^ h^−1^ [25].

**Figure 3:**
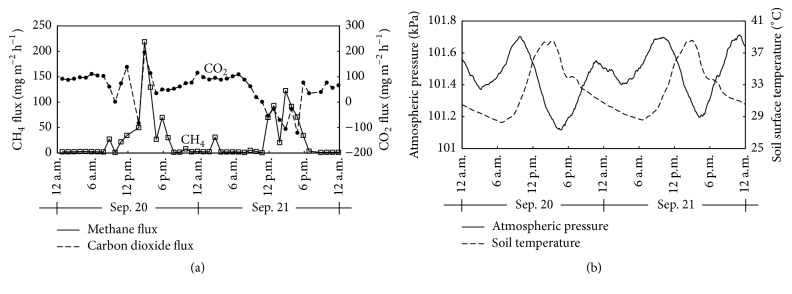
Temporal changes on September 20 and 21 in CH_4_ and CO_2_ fluxes measured with the automatic closed chamber method (a) and atmospheric pressure and soil surface temperature (b).

**Figure 4:**
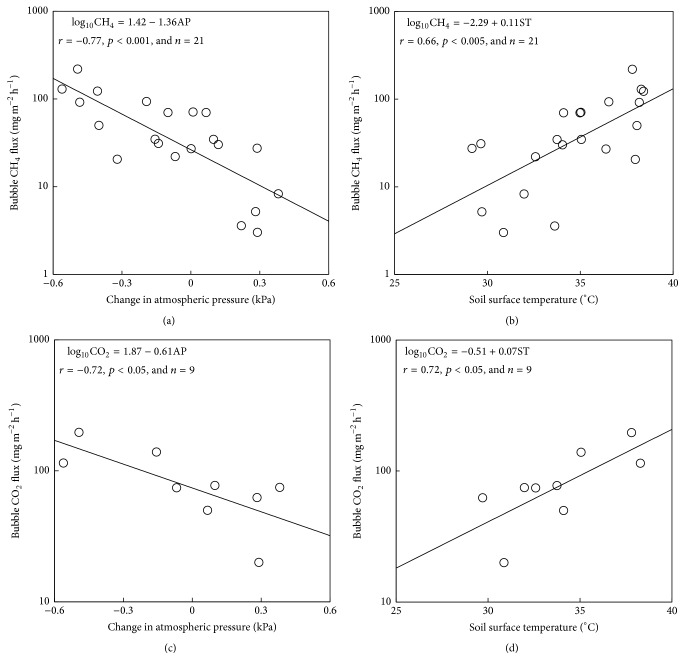
Relationship between CH_4_ emission by bubble ebullition and change of atmospheric pressure (a) or soil surface temperature (b). Relationship between CO_2_ emission by bubble ebullition and change of atmospheric pressure (c) or soil surface temperature (d). The change in atmospheric pressure was determined as the difference between the local maximum or minimum value and the value closest to the time when the CH_4_ or CO_2_ ebullition occurred.

The large CH_4_ emissions via bubble ebullition mainly occurred between 10:00 a.m. and 5:00 p.m. local time (Figure 3(a)). During this period, atmospheric pressure markedly decreased and reached a minimum value (Figure 3(b)). A night-time CH_4_ ebullition event also occurred at 2:50 a.m. local time on September 21 (Figures 2(c), 3(a), and 3(b)), once again when air pressure decreased. There was a significant negative linear correlation between atmospheric pressure and log_10_-CH_4_ emission by bubble ebullition (Figure 4; *r* = −0.77, *p* < 0.001). These results suggesting that decreases in atmospheric pressure triggered the CH_4_ ebullitions in the tropical rice paddy field are supported by the findings of Tokida et al. [18, 19] who reported that decreases in atmospheric pressure triggered CH_4_ ebullitions in peatlands.

In peatlands, air pressure reduction expands bubble volume and thereby enhances bubble buoyancy which causes the bubbles to rise to the water surface [16]. Reduced air pressure also increases the CH_4_ concentration of gas bubbles by degassing dissolved CH_4_ in soil solution [16, 26, 27]. These factors probably caused the higher CH_4_ emissions via ebullition that were found in the current study. Moreover, the higher CH_4_ ebullition emissions in the daytime, compared with nighttime, are probably due to larger decreases in daytime atmospheric pressure which would increase the volume of the bubbles and the CH_4_ concentration.

Rising soil temperature also increases the buoyancy and CH_4_ concentration of bubbles as barometric pressure decreases [17, 27]. In the current study, soil surface temperature increased from around 6:30 a.m. and reached a maximum value at 3:00–3:30 p.m. on each day (Figure 3(b)). This period approximately corresponded to that when CH_4_ ebullition events frequently occurred. The positive and significant correlation between soil surface temperature and log_10_-CH_4_ emission via bubble ebullition (*r* = 0.66; *p* < 0.005; Figure 4(b)) indicates that the increase in soil surface temperature contributed to CH_4_ ebullitions in the daytime. Ebullition events occurred at 8:50 a.m. on both days and at 9:50 a.m. on September 21, even though atmospheric pressure did not fall between 6:30 a.m. and 10:00 a.m. on either day. These ebullitions indicate that the rising soil temperature principally triggered the release of bubbles at those times. Rising soil temperature also has a role in enhancing methanogenic activities, leading to increases in CH_4_ production in soil [28]. Therefore such increased biological activities might have also increased the CH_4_ concentration in the bubbles.

CH_4_ emission via bubble ebullition (546–617 mg m^−2^ d^−1^) contributed 95-96% of total daily CH_4_ emission (567–647 mg m^−2^ d^−1^) through flooded water (Table 1). These CH_4_ ebullitions mainly occurred in the daytime and were associated with falling atmospheric pressure and increasing soil temperature, as discussed above (Figures 3(a) and 3(b)). In contrast, CH_4_ emission by diffusion (21–30 mg m^−2^ d^−1^) accounted for only 3.7–4.7% of total daily CH_4_ emission from flooded water (Table 1). The CH_4_ emissions by diffusion were mostly observed at nighttime when soil temperature decreased (Figures 3(a) and 3(b)). Therefore, these results clearly show that CH_4_ emission in rice paddy fields is predominant by daytime ebullition from flooded water with much lower CH_4_ emissions at nighttime by diffusion.

### 3.2. CO_2_ Emission

Episodic increases in CO_2_ concentration were found in 14 of the 21 measurements when CH_4_ ebullition events occurred. During these 14 chamber closure periods, the CO_2_ concentration in the chamber air increased abruptly (Figures 2(b) and 2(f)) at about the same time as CH_4_ concentration increased (Figures 2(a) and 2(e)). These similar patterns indicate that CO_2_ was released to the atmosphere in the bubbles along with the CH_4_. In the other 7 measurements, there was a steady increase in CO_2_ concentration but no episodic increase, as shown in Figure 2(d), while CH_4_ concentration abruptly increased (Figure 2(c)). This suggests these bubbles did not contain much CO_2_.

CO_2_ uptake via the photosynthetic activities of the aquatic plants was also observed in these measurements. In the other 25 measurements, there was a transfer of CO_2_ from flooded water to the atmosphere by diffusion, likely due to the gradient in CO_2_ concentration at the interface between the flooded water and the atmosphere and also due to respiration of the aquatic plants [21]. The values of CO_2_ fluxes ranged between −120.4 and 196.2 mg m^−2^ h^−1^ which are within the previously reported range of −285.1 to 459.4 mg m^−2^ h^−1^ [29].

On September 20, most of the CO_2_ fluxes were outgoing emissions due to bubble ebullitions. The highest CO_2_ emission (196.2 mg m^−2^ h^−1^) occurred at 2:50 p.m. (Figures 2(d) and 3(a)), coinciding with a high CO_2_ concentration in the bubbles of up to 11.74% (v/v). However, at 1:50 p.m., there was a negative (incoming) CO_2_ flux, even though there was a CO_2_ ebullition event. This overall negative flux must have been due to the fact that CO_2_ uptake by photosynthesis of the aquatic plants exceeded emissions by bubble ebullition, as shown in Figure 2(f) for CO_2_ transfer.

During the daytime on September 21, the CO_2_ fluxes mainly showed negative values even though CO_2_ ebullition events were observed. Therefore, this indicates that CO_2_ assimilation by the aquatic plants dominated CO_2_ fluxes on that day.

The log_10_-CO_2_ emissions by bubble ebullitions, omitting measurements with evidence of absorption by plant photosynthesis, were significantly correlated to changes in atmospheric pressure (*r* = −0.72; *p* < 0.05; Figure 4(c)) and soil surface temperature (*r* = 0.72; *p* < 0.05; Figure 4(d)). This indicates that these two environmental factors control CO_2_ ebullition in addition to CH_4_ ebullition. As previously discussed, these two triggered expanding bubble volume and degassing of gas dissolved in soil solution [16, 17]. In addition, the soil surface temperature was between 27 and 40°C during the measuring period which was optimal for respiratory soil microbes in the submerged paddy soil [28]. Therefore, all these factors probably enhanced CO_2_ bubble ebullitions.

CO_2_ emission by bubble ebullition, accounted for only 13–35% of total CO_2_ emissions, compared with 65–87% from CO_2_ diffusion (Table 2), indicating that CO_2_ ebullition did not dominate CO_2_ emissions from flooded water unlike CH_4_ ebullition. This is probably due to the fact that CO_2_ uptake by aquatic plants would have exceeded CO_2_ emission by bubble ebullition. Moreover, the very low concentration of CO_2_ in the bubbles also contributed to lower CO_2_ emission by bubble ebullition. The CO_2_ emissions by diffusion mostly occurred at nighttime just like for CH_4_. The nighttime CO_2_ emissions by diffusion were mostly attributed to the gradient in CO_2_ concentrations between the atmosphere and the flooded water and also to CO_2_ respiration by small aquatic plants [21].

## 4. Conclusions

Our study found that daytime CH_4_ ebullition events in tropical rice paddy fields occurred due to falling atmospheric pressure and increasing soil surface temperature. At nighttime, the drop in atmospheric pressure predominately triggered the CH_4_ ebullition because soil temperature was low compared with that in the daytime. The fact that CH_4_ and CO_2_ concentrations in the chamber air increased abruptly when bubbles were released suggests that bubble ebullition events caused not only CH_4_ emission but also CO_2_ emission. The CO_2_ ebullition events were also controlled by decreases in air pressure and increases in soil temperature. Therefore, diurnal changes in atmospheric pressure and soil temperature play major roles in regulating CH_4_ and CO_2_ ebullitions in tropical rice paddy fields.

We also found that CH_4_ emission was predominant due to daytime ebullition, whereas only a small proportion of CO_2_ emissions was due to daytime ebullition. The low CO_2_ ebullition throughout the day was due to CO_2_ photosynthesis and respiration by aquatic plants, meaning that CO_2_ emission was mainly by diffusion between flooded water and the atmosphere.

## Figures and Tables

**Table 1:** Cumulative CH_4_ emissions and relative contribution of bubble ebullition and diffusion processes to total emissions.

Date	CH_4_ ebullition (mg m^−2^ d^−1^)	Via CH_4_ ebullition (%)	CH_4_ diffusion (mg m^−2^ d^−1^)	Via CH_4_ diffusion (%)	Total CH_4_ emission (mg m^−2^ d^−1^)
Sep. 20	617.4	95.3	30.3	4.7	647.7
Sep. 21	546.2	96.3	20.9	3.7	567.1

**Table 2:** Cumulative CO_2_ emissions and relative contributions of bubble ebullition and diffusion processes to total emissions.

Date	CO_2_ ebullition (mg m^−2^ d^−1^)	Via CO_2_ ebullition (%)	CO_2_ diffusion (mg m^−2^ d^−1^)	Via CO_2_ diffusion (%)	Total CO_2_ emission (mg m^−2^ d^−1^)
Sep. 20	648.2	35.0	1203.8	65.0	1852.0
Sep. 21	159.7	13.3	1040.4	86.7	1200.1
